# Activity of Platinum-Based Chemotherapy in Patients With Advanced Prostate Cancer With and Without DNA Repair Gene Aberrations

**DOI:** 10.1001/jamanetworkopen.2020.21692

**Published:** 2020-10-28

**Authors:** Sabine Schmid, Aurelius Omlin, Celestia Higano, Christopher Sweeney, Nieves Martinez Chanza, Niven Mehra, Malou C. P. Kuppen, Himisha Beltran, Vincenza Condeduca, Daniel Vargas Pivato de Almeida, Fernando Cotait Maluf, William K. Oh, Che-Kai Tsao, Oliver Sartor, Elisa Ledet, Giuseppe Di Lorenzo, Steven M. Yip, Kim N. Chi, Diletta Bianchini, Ugo De Giorgi, Aaron R. Hansen, Tomasz M. Beer, Lavaud Pernelle, Rafael Morales-Barrera, Marcello Tucci, Elena Castro, Kostas Karalis, Andries M. Bergman, Mo Linh Le, Ursina Zürrer-Härdi, Carmel Pezaro, Hiroyoshi Suzuki, Andrea Zivi, Dirk Klingbiel, Sämi Schär, Silke Gillessen

**Affiliations:** 1 Department of Medical Oncology and Haematology, Cantonal Hospital of St Gallen, St Gallen, Switzerland; 2 Division of Medical Oncology and Hematology, Princess Margaret Cancer Centre, Toronto, Canada; 3 Seattle Cancer Care Alliance, University of Washington, Seattle; 4 Department of Medical Oncology, Dana Farber Cancer Institute, Boston, Massachusetts; 5 Radboud University, Medical Center Nijmegen, Utrecht, the Netherlands; 6 Institute for Medical Technology Assessment, Erasmus School of Health Policy and Management, Erasmus University, Rotterdam, the Netherlands; 7 Department of Medical Oncology, Weill Cornell Medicine, New York, New York; 8 Istituto Scientifico Romagnolo per lo Studio e la Cura dei Tumori IRCCS, Meldola, Italy; 9 Department of Medical Oncology Beneficencia Portuguesa de São Paulo, São Paulo, Brazil; 10 Sloan Kettering Institute, Memorial Sloan Kettering Cancer Center, New York, New York; 11 Department of Medical Oncology, Hospital Israelita Albert Einstein, Beneficencia Portuguesa de São Paulo, São Paulo, Brazil; 12 Oncoclinicas Oncology Group, Brasilia, Brazil; 13 Division of Hematology and Medical Oncology, Tisch Cancer Institute, Icahn School of Medicine, Mount Sinai Hospital, New York, New York; 14 Tulane Cancer Center, Tulane Medical School, New Orleans, Louisiana; 15 Medical Oncology, Department of Medicine and Health Sciences Vincenzo Tiberio, University of Molise, Campobasso, Italy; 16 British Columbia Cancer, Vancouver, Canada; 17 Division of Clinical Studies, Prostate Cancer Targeted Therapies Group, Institute of Cancer Research, Royal Marsden NHS Foundation Trust, Sutton, United Kingdom; 18 Maidstone Hospital, Kent, United Kingdom; 19 Oregon Health and Science Knight Cancer Institute, Oregon Health and Science University, Portland; 20 Department of Cancer Medicine, Gustave Roussy, Cancer Campus, Grand Paris, Université Paris-Sud, Université Paris-Saclay, Villejuif, France; 21 Department of Medical Oncology, Vall d’Hebron University Hospital, Barcelona, Spain; 22 Division of Medical Oncology, San Luigi Gonzaga Hospital, Department of Oncology, University of Turin, Orbassano, Turin, Italy; 23 Prostate Cancer Clinical Research Unit, Spanish National Cancer Research Centre, Madrid, Spain; 24 Department of Genitourinary Medical Oncology, Athens Medical Center, Athens, Greece; 25 Division of Internal Medicine and Oncogenomics, Netherlands Cancer Institute, Amsterdam, the Netherlands; 26 Guy’s and St Thomas’ Hospital, London, United Kingdom; 27 Department of Medical Oncology, Cantonal Hospital Winterthur, Winterthur, Switzerland; 28 Department of Oncology, Eastern Health, Box Hill, Victoria, Australia; 29 Department of Urology, Toho University Sakura Medical Center, Chiba, Japan; 30 Department of Medical Oncology, Azienda Ospedaliera Universitaria Integrata di Verona, Verona, Italy; 31 Section of Cancer, Department of Surgery and Cancer, Faculty of Medicine, Imperial College London, London, United Kingdom; 32 Coordinating Center, Swiss Group for Clinical Cancer Research, Bern, Switzerland; 33 Oncology Institute of Southern Switzerland, Bellinzona, Switzerland; 34 Faculty of Biomedical Sciences, Università della Svizzera Italiana, Lugano, Switzerland

## Abstract

**Importance:**

DNA repair gene aberrations occur in 20% to 30% of patients with castration-resistant prostate cancer (CRPC), and some of these aberrations have been associated with sensitivity to poly(ADP-ribose) polymerase (PARP) inhibition platinum-based treatments. However, previous trials assessing platinum-based treatments in patients with CRPC have mostly included a biomarker-unselected population; therefore, efficacy in these patients is unknown.

**Objective:**

To characterize the antitumor activity of platinum-based therapies in men with CRPC with or without DNA repair gene alterations.

**Design, Setting, and Participants:**

In this case series, data from 508 patients with CRPC treated with platinum-based therapy were collected from 25 academic centers from 12 countries worldwide. Patients were grouped by status of DNA repair gene aberrations (ie, cohort 1, present; cohort 2, not detected; and cohort 3, not tested). Data were collected from January 1986 to December 2018. Data analysis was performed in 2019, with data closure in April 2019.

**Exposure:**

Treatment with platinum-based compounds either as monotherapy or combination therapy.

**Main Outcomes and Measures:**

The primary end points were as follows: (1) antitumor activity of platinum-based therapy, defined as a decrease in prostate-specific antigen (PSA) level of at least 50% and/or radiological soft tissue response in patients with measurable disease and (2) the association of response with the presence or absence of DNA repair gene aberrations.

**Results:**

A total of 508 men with a median (range) age of 61 (27-88) years were included in this analysis. DNA repair gene aberrations were present in 80 patients (14.7%; cohort 1), absent in 98 (19.3%; cohort 2), and not tested in 330 (65.0%; cohort 3). Of 408 patients who received platinum-based combination therapy, 338 patients (82.8%) received docetaxel, paclitaxel, or etoposide, and 70 (17.2%) received platinum-based combination treatment with another partner. A PSA level decrease of at least 50% was seen in 33 patients (47.1%) in cohort 1 and 26 (36.1%) in cohort 2 (*P* = .20). In evaluable patients, soft tissue responses were documented in 28 of 58 patients (48.3%) in cohort 1 and 21 of 67 (31.3%) in cohort 2 (*P* = .07). In the subgroup of 44 patients with *BRCA2 *gene alterations, PSA level decreases of at least 50% were documented in 23 patients (63.9%) and soft tissue responses in 17 of 34 patients (50.0%) with evaluable disease. In cohort 3, PSA level decreases of at least 50% and soft tissue responses were documented in 81 of 284 patients (28.5%) and 38 of 185 patients (20.5%) with evaluable disease, respectively.

**Conclusions and Relevance:**

In this study, platinum-based treatment was associated with relevant antitumor activity in a biomarker-positive population of patients with advanced prostate cancer with DNA repair gene aberrations. The findings of this study suggest that platinum-based treatment may be considered an option for these patients.

## Key Points

**Question:**

Is there a role for platinum-based treatment in molecularly selected patients with advanced prostate cancer?

**Findings:**

In a case series of 508 patients, platinum-based therapy was associated with antitumor activity, especially among patients with known DNA repair gene aberrations. In patients with DNA repair gene aberrations, nearly half had a decrease in prostate-specific antigen levels of at least 50% and experienced soft tissue responses.

**Meaning:**

In patients with prostate cancer and DNA repair gene aberrations, platinum-based therapy may be considered a treatment option.

## Teaser

This case series characterizes the antitumor activity of platinum-based therapies in men with castration-resistant prostate cancer with or without DNA repair gene alterations.

## Introduction

Despite significant progress in drug development for advanced prostate cancer patients in the past decade, new active compounds, ideally for molecularly selected patients, are urgently needed.^1,2^ Platinum-based compounds have been evaluated in clinical trials and used clinically as monotherapy or in combination with other chemotherapy agents mainly in the setting of castration-resistant disease but also in hormone-sensitive disease.^3,4^ A phase 3 clinical trial of satraplatin compared with prednisone^5^ demonstrated an improvement in progression-free survival and pain control, but no overall survival benefit was seen in unselected patients.

Platinum chemotherapy activity has been associated with its ability to crosslink with purine bases in DNA, interfering with DNA repair mechanisms and causing DNA damage and apoptosis. In various cancer types, responses are enhanced in the presence of underlying double-strand DNA repair alterations in the tumor, resulting in synthetic lethality.^6^ In triple-negative breast cancer (TNBC), carboplatin is highly effective in patients with known *BRCA1* (OMIM 113705) and *BRCA2* (OMIM 600185) tumors,^7^ and 2 phase 3 trials have shown benefit of poly(ADP-ribose) polymerase (PARP) inhibitors in patients with TNBC and germline *BRCA* variations.^8^ The role of these agents in an unselected TBNC population is controversial; however, there is evidence that some patients with variations in homologous recombination genes other than *BRCA1* and *BRCA1* (germline or somatic) can derive benefit from platinum-based treatment.^9^ On the other hand, alterations in other non–homologous recombination DNA damage response genes, such as *PTEN *(OMIM 601728), are not associated with response to the same extent.^9^

Genomic aberrations that impair DNA repair genes occur at a frequency of up to 20% to 30% in advanced prostate cancer.^10,11,12^ Some of these aberrations, which can either be found as germline or somatic alterations in homologous recombination DNA repair genes or DNA damage checkpoints, have been associated with sensitivity to platinum compounds and/or PARP inhibition in preclinical studies and in clinical trials.^13,14,15,16,17^

Three published case series,^18,19,20^ which included a total of 14 patients with metastatic prostate cancer with DNA repair gene aberrations treated with platinum-based chemotherapy, reported encouraging antitumor activity. The recently presented prospective phase 3 PROFOUND study evaluating the PARP-inhibitor olaparib in molecularly selected patients with advanced prostate cancer harboring DNA repair gene aberrations has shown a significant benefit in radiographic progression-free survival and overall response rate for olaparib compared with the sequential use of abiraterone or enzalutamide, thereby strengthening the previously reported findings of the TOPARP study.^21,22,23^ Data from these trials suggest higher antitumor activity in patients with *BRCA2* alterations.^22^

Through the collaborative efforts of an international consortium, we identified a large series of patients with castration-resistant prostate cancer (CRPC) who were treated with platinum-based chemotherapy. Using these data, we performed a retrospective analysis to characterize the antitumor activity (ie, decrease in prostate-specific antigen [PSA] level, radiographic response, and time receiving treatment) of platinum-based therapies (monotherapy and/or combination) in men with CRPC with or without DNA repair gene alterations.

## Methods

### Patients

Patients with biochemically or histologically confirmed advanced prostate cancer (metastatic or locally advanced and not amenable to locoregional treatment with curative intent) treated with a platinum compound (cisplatin or carboplatin) either as monotherapy or as part of combination chemotherapy were eligible for this analysis. Patients with primary pure small cell carcinoma of the prostate or insufficient data for analysis of the primary end point were excluded. Clinical data from 25 cancer centers from 12 countries worldwide were collected by local investigators. Investigators were encouraged to include all patients eligible at their site for the analysis. Approval of the local ethics committee was obtained before data collection, and informed consent was obtained depending on local regulations. Clinical data were collected locally at each center and assembled in an electronic master database at the coordinating center. Quality control was assured by queries in cases of nonplausible data and inconsistencies; however, no review of the source documentation was performed. The the reporting guideline for case series was followed.^24^

The primary outcome measure was evaluation of antitumor activity (decrease in PSA level and soft tissue response) to platinum-based therapy (monotherapy and/or combination therapies) and association of response with the presence or absence of DNA repair gene aberrations in patients with advanced prostate cancer. Local assays for assessment of DNA repair gene aberrations on tumor tissue or circulating DNA (ctDNA) were used. DNA repair gene alterations were defined as deleterious variations, such as protein truncating variations, splice site variations, deleterious missense variation, and homozygous deletions, in genes involved in the repair of DNA damage.

The decrease in PSA level is reported as per Prostate Cancer Working Group Criteria,^25^ with percentage change from baseline (increase or decrease) at 12 weeks and, separately, the maximal change (increase or decrease) at any time using a waterfall plot. Clinically significant PSA changes are defined as decreases of at least 50%. Soft tissue response was defined according to the Response Evaluation Criteria in the Solid Tumors guidelines version 1.1, with response being defined as an at least 30% decrease in the sum of the longest diameter of all target lesions (maximum of 2 lesions per organ and 5 lesions total), taking the baseline sum as reference. Tumor assessments were performed by local investigators. Time on platinum-based treatment was defined as time from start of platinum-based treatment to progression (defined as the end of treatment for clinical or radiological progressive disease or other reasons) or death; overall survival (OS) was defined as time from start of platinum-based treatment to death or last contact. Patients not experiencing an event were censored at the time of data cutoff (ie, September 24, 2019) or at last contact.

### Statistical Analysis

Continuous data were summarized by median, minimum, and maximum values. Categorical data were presented as absolute numbers and percentages. Time to event end points were assessed by the Kaplan-Meier method and are presented as median and interquartile range (IQR). The number of missing data points is given for all analyses. Frequency counts of categorical data in subgroups were statistically compared by Fisher exact tests. Between subgroup comparisons of numerical data were carried out by Kruskal-Wallis tests. Time to event end points were compared between subgroups using the log-rank test. The a priori significance level was *P* < .05, and all statistical tests were 2 sided. All analyses were done in R version 3.5.0 (R Project for Statistical Computing).

To simultaneously assess several factors that could be associated with OS, we performed a multivariable analysis using a Cox proportional hazards model (SAS version 9.4 [SAS Institute]), including clinically relevant and established prognostic factors, ie, age, presence of visceral metastases, presence or absence of DNA repair gene aberrations, treatment line, and type of platinum-based treatment (combination vs monotherapy) as independent variables.

## Results

**Figure 1.**
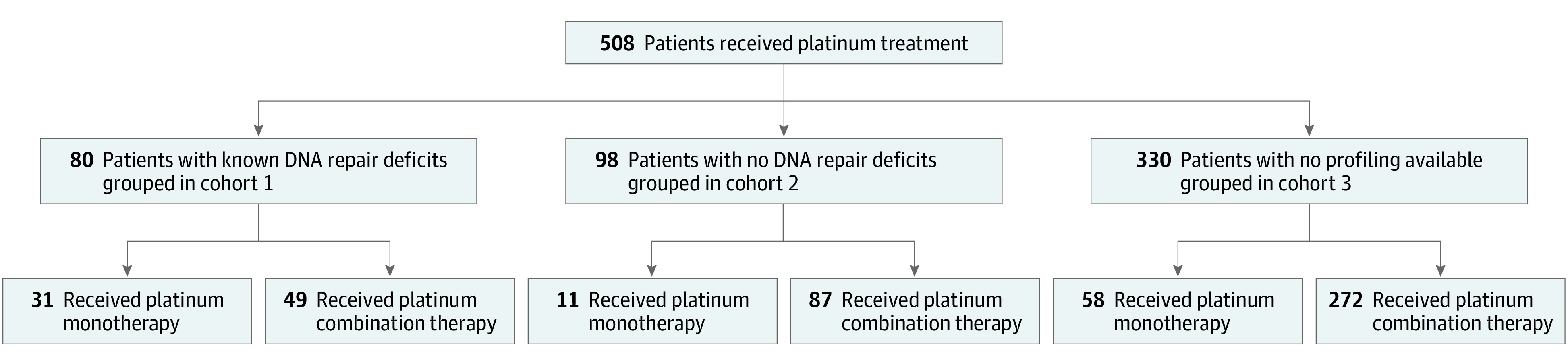
Study Flow Diagram

A total of 508 men who were diagnosed with prostate cancer between 1986 and 2018 were included in the study (median [range] age, 61 [37-88 ] years; baseline median [range] PSA level, 18.5 ng/mL [0.7-7577 ng/mL] [to convert to micrograms per liter, multiply by 1.0]). Overall, 216 (42.5%) had de novo metastatic disease at diagnosis and treated with platinum-based chemotherapy between 1999 and 2019. A total of 178 patients (35.0%) had molecular profiling, with DNA repair gene aberrations detected in 80 patients (15.7%; cohort 1) and no aberrations detected in 98 patients (19.3%; cohort 2). In 330 patients (65.0%), tumor genomic profiling was not performed (cohort 3) (Figure 1).

Patients with known DNA repair gene aberrations, compared with patients without DNA repair gene aberrations, had higher median (range) PSA levels at diagnosis (33.0 [2.1-1759.0] ng/mL vs 17.5 [1.1-1530.0] ng/mL, *P* = .20), higher rates of de novo metastatic disease (42 [52.5%] vs 40 [40.8%], *P* = .10) (eTable 1 in the Supplement), and a shorter median (IQR) time from diagnosis of CRPC to start of platinum-based treatment (32.7 [11.8-80.6] months vs 52.5 [20.4-89.2] months; *P* = .06), although none of the differences were statistically significant. Nearly 70% of patients (341 [67.1%]) had received at least 1 prior line of systemic therapy before platinum-based chemotherapy was started. Of note, a high percentage of patients with visceral metastases (cohort 1, 44 [55.0%]; cohort 2, 57 [58.2%]) at the start of platinum-based therapy were included.

DNA repair gene aberrations were detected by various methods: 27 patients (33.8%), fresh biopsy material; 24 (30.0%), archival tissue; 22 (27.5%), ctDNA; and 7 (8.8%), unknown methods. The most common aberrations included alterations in *BRCA2* (44 [55.0%]), *ATM* (OMIM 607585; 12 [15.0%]), and *BRCA1* (3 [3.8%]). These were somatic alterations in 57 patients (71.3%). Germline alterations were found in 23 patients (28.8%) and were mainly *BRCA2* (17 [73.9%]), *BRCA1* (2 [8.7%]), and *ATM* (3 [13.0%]). Baseline characteristics depending on type of DNA repair gene aberration are summarized in eTable 2 in the Supplement.

Platinum-based therapy was given as first-line CRPC treatment in 167 patients (32.8%), second-line treatment in 145 patients (28.5%), and third-line treatment in 68 patients (13.4%) (Table 1). The percentage of patients receiving first-line platinum treatment was comparable between all cohorts, and in most cases, combination therapy was chosen (139 [83.2%]). Most patients (408 [80.3%]) received a platinum-combination treatment. Combination chemotherapy drugs used included docetaxel (180 [44.1%]), etoposide (92 [22.5%]), and paclitaxel (66 [16.2%]). Only 100 patients (19.7%) were treated with platinum-based monotherapy (88 [88.0%] with carboplatin); however, the proportion was higher in cohort 1 compared with cohort 2 (31 [38.8%] vs 11 [11.2%]; *P* < .001).

**Table 1.** Characteristics of Study Participants at Start of Platinum-Based Chemotherapy

Characteristic	Patients, No. (%)			*P* value	
	Cohort 1, with DNA repair gene aberrations(n = 80)	Cohort 2, no DNA repair gene aberrations (n = 98)	Cohort 3, not assessed (n = 330)	All cohorts	Cohorts 1 vs 2
**Baseline characteristics at start of platinum therapy**					
Age, median (range), y	65 (38-81)	67 (49-86)	67 (42-90)	.006	.02
Missing. No.	0	0	2		
PSA level, median (range), ng/mL	120 (0.02-4124)	90.2 (0.05-3030)	136.6 (0.01-9145)	.04	.05
Missing, No.	5	14	16		
Alkaline phosphatase level, median (range), U/L	161.5 (44-1661)	159 (30-1260)	140 (12-3870)	.50	.50
Missing, No.	10	15	75		
Lactate dehydrogenase level, median (range), U/L	327 (12-2680)	282.5 (9-6714)	330.5 (131-5432)	.20	.30
Missing, No.	18	24	154		
Hemoglobin leve, median (range)l, g/dL	11.0 (7.0-15.0)	10.9 (7.5-15.0)	10.9 (6.0-15.6)	.80	.90
Missing, No.	11	13	55		
Albumin level, median (range), g/dL	3.4 (1.9-4.3)	3.3 (2.0-4.3)	3.6 (2.0-6.6)	.10	.80
Missing, No.	17	26	125		
Opiates					
No	26 (41.3)	30 (35.7)	94 (33.3)	.04	.01
Strong opioid	30 (47.6)	35 (41.7)	136 (48.2)		
Weak opioid	7 (11.1)	19 (22.6)	52 (18.4)		
Missing, No.	17	14	48		
Time from diagnosis to platinum based chemotherapy, median (IQR), mo	33 (12-81)	53 (20-89)	65 (28-100)	<.001	.06
Missing, No.	7	0	7		
Distribution of metastases at start of platinum based treatment					
Bone metastases	66 (82.5)	89 (90.8)	295 (89.4)	.20	.20
Lymph node metastases	55 (68.8)	55 (56.1)	231 (70)	.02	.09
Visceral metastases	44 (55)	57 (58.2)	173 (52.4)	.70	.80
Missing, No.	1	0	5	NA	NA
**Platinum therapy**					
Platinum monotherapy					
Overall	31 (38.8)	11 (11.2)	58 (17.6)	<.001	<.001
Carboplatin, No./total No. (%)	30/31 (96.8)	8/11 (72.7)	50/58 (86.2)		
Cisplatin, No./total No. (%)	1/31 (3.2)	3/11 (27.3)	8/58 (13.8)		
Platinum combination therapy					
Overall	49 (61.3)	87 (88.8)	272 (82.4)	<.001	<.001
Carboplatin, No./total No. (%)	38/49 (77.6)	73/87 (83.9)	252/272 (92.6)		
Cisplatin, No./total No. (%)	11/49 (22.4)	14/87 (16.1)	18/272 (6.6)		
Oxaliplatin, No./total No. (%)	0	0	2/272 (0.7)		
Combination partner					
Docetaxel, No./total No. (%)	17/49 (34.7)	24/87 (27.6)	139/272 (51.1)	<.001	.090
Etoposide, No./total No. (%)	13/49 (26.5)	31/87 (35.6)	48/272 (17.6)		
Other, No./total No. (%)	16/49 (32.7)	17/87 (19.5)	37/272 (13.6)		
Paclitaxel, No./total No. (%)	3/49 (6.1)	15/87 (17.2)	48/272 (17.6)		
Prior treatment lines					
0	25 (31.2)	36 (36.7)	106 (32.1)	<.001	.80
1	16 (20)	15 (15.3)	114 (34.5)		
2	14 (17.5)	18 (18.4)	36 (10.9)		
≥3	25 (31.2)	29 (29.6)	74 (22.4)		

Abbreviations: IQR, interquartile range; NA, not applicable; PSA, prostate specific antigen.

SI conversion factors: To convert albumin and hemoglobin to grams per liter, multiply by 10; alkaline phosphatase and lactate dehydrogenase to microkatals per liter, multiply by 0.0167; and PSA to micrograms per liter, multiply by 1.0.

### Outcomes of Cohort 1 vs Cohort 2

**Figure 2.**
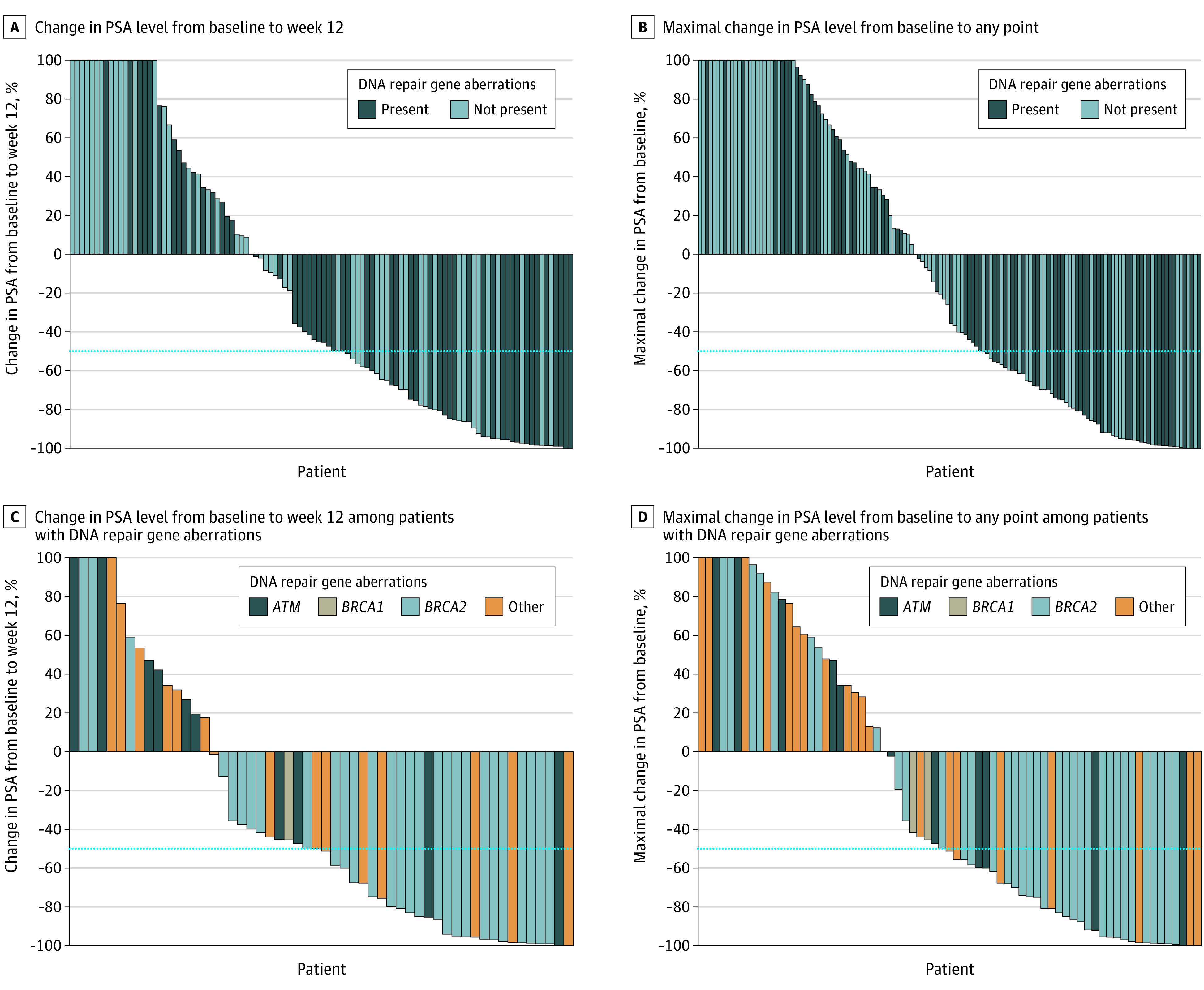
Prostate-Specific Antigen (PSA) Level Changes From Baseline to Week 12 and Maximal PSA Change to Any Time Point

A PSA level decrease of at least 50% was seen in 33 patients (47.1%) in cohort 1 vs 26 (36.1%) in cohort 2 (*P* = .20) (Table 2 and Figure 2A). A soft tissue response was seen in 28 patients (48.3%) in cohort 1 vs 21 (31.3%) in cohort 2 (*P* = .07) (Table 2).

**Table 2.** Antitumor Activity of Platinum Chemotherapy in Cohorts 1 vs 2 and in Subgroups of Patients With DNA Repair Gene Aberrations

Outcome	Patients with DNA repair gene aberrations, No. %		*P* value, cohort 1 vs 2	Patients with DNA repair gene aberrations, No. %				*P* value, among subgroups
	Cohort 1, yes (n = 80)	Cohort 2, no (n = 98)		*BRCA2* (n = 44)	*BRCA1* (N = 3)	*ATM* (N = 12)	Other (N = 21)	
PSA level decrease of ≥50% on platinum therapy	33 (47.1)	26 (36.1)	.20	23 (63.9)	0	4 (36.4)	6 (28.6)	.02
Missing, No.	10	26		8	1	1	0	
Soft tissue response on platinum therapy	28 (48.3)	21 (31.3)	.07	17 (50.0)	0	2 (28.6)	9 (56.3)	.60
Missing, No.	22	31		10	2	5	5	
Time receiving treatment and survival, median (IQR), mo	3.4 (1.6-6)	2.8 (1.7-4.6)	.30	7.1 (3.7-13)	3.2 (0-NA)	4.7 (2.2-10)	2.8 (1.8-NA)	.20
Missing, No.	0	1		0	0	0	0	
OS from start of platinum therapy, median (IQR), mo	14 (5.7-34)	9.2 (5.5-19)	.20	15 (10-34)	4.1 (3.8-4.4)	9.3 (6.5-11)	4.9 (3.6-NA)	.04
Missing, No.	0	0		0	0	0	0	

Abbreviations: IQR, interquartile range; OS, overall survival; PSA, prostatic-specific antigen.

Patient groups with different DNA repair gene aberrations had different proportions of PSA level decreases of at least 50% (*BRCA2*, 23 of 44 [63.9%]; *BRCA1*, 0 of 3; *ATM*, 4 of 11 [36.4%]; other, 6 of 21 [28.6%]; *P* = .02) (Figure 2C); however, no significant difference in soft tissue responses was observed, irrespective of type of DNA repair gene aberration (Table 2). In the 12 patients with ATM alterations, a PSA decline of at least 50% was documented in 4 patients (33.3%) and a soft tissue response in 2 of 7 patients (28.6%) with evaluable disease.

Median (IQR) treatment duration on platinum-based treatment was 3.0 (1.7-4.6) months without relevant differences between cohorts (Table 2; eFigure 1 in the Supplement). Seven patients (8.8%) with known DNA repair alterations had PARP therapy before platinum treatment. Of these patients, only 1 (14.3%) had a documented PSA decrease of at least 50% on platinum-based chemotherapy.

**Figure 3.**
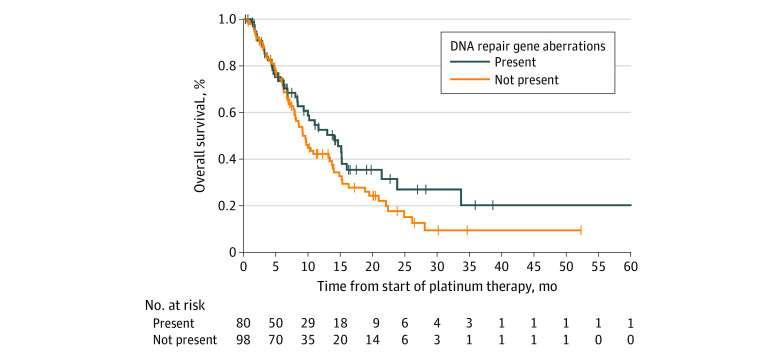
Overall Survival Among Patients With and Without DNA Repair Gene Aberrations Receiving Platinum-Based Therapy

Median (IQR) OS from start of platinum-based therapy was lower in patients without DNA repair gene aberrations compared with patients with known DNA repair gene aberrations (9.2 [5.5-19.5] months vs 14.1 [5.7-33.7] months), although this did not reach statistical significance (*P* = .20) (Figure 3; Table 2).

Median OS differed significantly in cohorts with different DNA repair gene alterations; namely, median (IQR) OS from start of platinum-based therapy was 15.2 (9.9-33.7) months in patients with *BRCA2* alterations, 9.3 (6.5 to 11.0) months in patients with *ATM* alterations, 4.1 (3.8 to 4.4) months in patients with *BRCA1* alterations, and 4.9 (3.6 to not reached) in patients with other alterations (*P* = .04) (Table 2; eFigure 2 in the Supplement). In a multivariable Cox regression for OS, only type of platinum-treatment remained significant (hazard ratio, 0.52; 95% CI, 0.31-0.90; *P* = .01) (eTable 3 in the Supplement).

A total of 31 patients (38.8%) with known DNA repair gene aberrations and 11 patients (11.2%) without DNA repair gene aberrations were treated with platinum monotherapy. In patients with known DNA repair gene aberrations, 10 (32.3%) had a PSA level decrease of at least 50%. Soft tissue response was seen in 14 patients (45.2%) in cohort 1 with measurable disease. Response to monotherapy was lower in patients without DNA repair gene aberrations, with PSA level decreases of at least 50% and soft tissue response in 3 patients (27.3%) (eTable 4 in the Supplement). Median (IQR) OS was 6.4 (3.6-15.2) months in patients with known alterations and 6.7 (3.5-15.2) months in patients without alterations (eFigure 3 in the Supplement).

Overall, 49 patients (61.3%) with known DNA repair gene aberrations and 87 patients (88.8%) without DNA repair gene aberrations were treated with platinum-based combination therapy. In patients with known DNA repair gene aberrations, 23 (46.9%) had a decrease in PSA level of at least 50%. Soft tissue response was seen in 14 patients (28.6%) with measurable disease. Response to combination therapy was again lower in patients without DNA repair gene aberrations, with PSA level decreases of at least 50% in 23 patients (26.4%) and soft tissue response in 18 (20.7%) (eTable 4 in the Supplement). Median (IQR) OS was 15.2 (9.9-33.7) months in patients with known alterations and 9.8 (6.1-19.5) months in patients without alterations (eFigure 3 in the Supplement).

### Outcomes in Cohort 3

Patients in cohort 3 (ie, those without genomic profiling) had an overall PSA level decrease of at least 50% in 81 of 284 patients (28.5%) (eFigure 4 in the Supplement); soft tissue response was reported in 38 of 185 (20.5%) with evaluable disease. A total of 58 patients (17.6%) were treated with platinum-based monotherapy and 272 (82.4%) with platinum-based combination therapy, and detailed response data are summarized in eTable 5 in the Supplement.

Median (IQR) treatment duration on platinum-based treatment was 3.0 (1.7-4.6) months and comparable with cohorts 1 and 2 (eFigure 5 in the Supplement). Median (IQR) OS from start of platinum therapy was 10.0 (5.7-17.7) months (eFigure 6 in the Supplement).

## Discussion

In this multicenter retrospective analysis of 508 patients with CRPC, most of whom had received at least 1 prior line of therapy, we found encouraging antitumor activity for treatment with platinum-based therapies in the cohort of patients with tumors harboring DNA repair gene aberrations. Although we observed numerically higher rates of PSA level decreases and soft tissue responses in patients with DNA repair gene aberrations compared with those without, there was no statistically significant difference and no OS benefit. In the subgroup of 44 patients with *BRCA2* gene alterations, PSA level decreases of at least 50% were documented in 23 patients (63.9%) and soft tissue responses in 17 patients (38.6%) with evaluable disease. This series is unique because it is among the largest to date that evaluates patients with identified DNA repair gene aberrations who received a platinum-based chemotherapy, very few of whom had previously received treatment with a PARP inhibitor because use of these agents outside of clinical trials was very limited.

Historically, platinum compounds as monotherapy or combination therapy have been widely studied in prostate cancer.^26^ Besides the SPARC trial,^5^ the studies were mostly small, recruited a molecularly unselected patient population, and demonstrated only moderate antitumor activity. However, a subgroup of patients seemed to derive benefit,^3^ namely patients with aggressive variant adenocarcinoma of the prostate (with alterations in at least 2 of the following: *TP53* [OMIM 191170], *RB1* [OMIM 614041], and *PTEN*, detected by next-generation sequencing or immunohistochemistry).^27^ With emerging data of a meaningful prevalence of somatic and germline DNA repair gene aberrations in patients with advanced prostate cancer^28^ and activity of PARP inhibitors in these patients,^12^ interest in platinum-based treatment arose again, with the hypothesis of increased activity in this specific subpopulation of patients with prostate cancer, analogous to data from patients with TNBC. Generally, combination treatment should be recommended; however, monotherapy remains an option, and there is expert consensus from the 2019 advanced prostate cancer consensus conference for carboplatin with target area under the curve (AUC) of 5 to 6 every 3 weeks as a preferred regimen.^33^

Response to platinum-based monotherapy in patients with DNA repair gene aberrations in our cohort was comparable with the recently presented trials of PARP monotherapy. In the phase 2 TOPARP-B trial assessing 2 different dosing levels of olaparib in patients with DNA repair alterations,^21^ olaparib at 400 mg and 300 mg was associated with soft tissue response of 24.2% and 16.2% of patients, respectively, and a PSA level decrease of at least 50% in 37.0% and 30.2%, respectively. This is in line with the overall response rate of 43.9% with rucaparib in patients with measurable disease in the phase 2 TRITON trial.^29^ Even more importantly, the PROFOUND trial, evaluating olaparib vs physician’s choice of enzalutamide or abiraterone in patients with CRPC and known DNA repair gene aberrations progressing on prior new hormonal agent, demonstrated a median OS of 18.5 months with olaparib in the total population, and an objective response rate of 33% was reported for patients with *BRCA1*, *BRCA2*, or *ATM* alterations.^22,23^ Olaparib and rucaparib were approved by the US Food and Drug Administration in May 2020 for patients with advanced prostate cancer and germline or somatic homologous recombination repair gene mutations (rucaparib only for *BRCA1* or *BRCA2*).

Response to platinum-based combination treatment in our cohort was more favorable than platinum-based monotherapy, and in most cases, a taxane was chosen as the combination partner, which represents the current standard-of-care chemotherapy in the unselected advanced prostate cancer population. However, it seems that in our data set monotherapy was more often used in patients with known DNA repair gene aberrations, whereas in patients without alterations or unknown molecular status, combination therapy was preferred. Taxanes are widely used in advanced prostate cancer in different treatment settings and the combination of carboplatin and paclitaxel as first-line, second-line, or third-line chemotherapy in patients with CRPC. In 2019, Castro et al^30^ presented the results of a prospective cohort study investigating the association of germline DNA repair gene aberrations with CRPC outcomes. In their cohort, response to taxanes was not different between variation carriers and noncarriers; however, duration of response was shorter in carriers, and treatment sequencing seemed to be of importance.^30,31^ Therefore, activity of the taxane component has to be taken into some account when interpreting our efficacy data in patients receiving combination treatment.

Our results showed consistently higher response rates of platinum-based treatment in molecularly selected patients, even though these patients often received platinum-based monotherapy, which seems to be less active than combination therapy overall. Subgroups for specific types of DNA repair gene aberrations were too small to draw any definitive conclusions, but the response rate (PSA decrease and objective response rate) in patients with *BRCA2* alterations was encouraging. These results are in line with the PROFOUND results, in which treatment benefit was more pronounced in patients with *BRCA1*, *BRCA2*, and *ATM* alterations compared with alterations in other gene alterations.^22^ In the exploratory analysis of gene-by-gene radiographic progression-free survival, benefit in patients with *BRCA1* and *ATM* alterations was less pronounced compared with patients with *BRCA2* alterations. In the TRITON phase 2 trial, evaluating rucaparib in patients with DNA repair gene aberrations, radiographic and PSA responses were observed in only 2 of 19 patients (10.5%) with exclusively *ATM* alterations.^32^ The antitumor activity of platinum compounds in our small cohort of 12 patients with *ATM* alterations is noteworthy. Overall, different DNA repair gene aberrations are most likely distinct entities with varying responses to platinum as well as PARP inhibition; therefore, they cannot be collectively addressed. More research with prospective trials in molecular subgroups is needed to better characterize which patients might derive benefit.

Importantly, the activity of platinum-based treatment was also seen in the unselected cohort 3 (ie, patients were not tested for DNA repair gene aberrations). This population is possibly most reflective of the general CRPC population seen in many centers around the world, where testing for DNA repair gene aberrations may not be available in daily clinical practice.

### Limitations

Our analysis has several limitations, including the retrospective design and data collection, including missing data in a subset of patients. Furthermore, even though quality control was assured by the coordinating center by queries in the case of nonplausible or inconsistent data, no formal review process of the source documentation of each individual contributing center was performed. Also, there was no control of patients excluded from the database on the center level. The bias was minimized by including all patients treated with a platinum-based chemotherapy and not only patients with known DNA repair gene alteration. Radiographic responses (ie, soft tissue response) were assessed retrospectively by local investigators. Patient selection is also an important limitation because the use of a platinum-based chemotherapy, especially in the first-line CRPC setting, could reflect the availability of treatment options; clinical factors, such as the high rate of de novo metastatic disease; and the presence of visceral metastases and/or molecular features. DNA repair gene aberrations were assessed by local assays and were not standardized; therefore, the number of genes tested differed by center. Many patients received platinum-taxane combination treatment and the contribution of taxane and platinum to the response cannot be measured separately. The high number of first-line platinum-based combination treatments suggests that patients with aggressive features were included; of note, we excluded patients with de novo pure small cell carcinoma from the analysis. Molecular profiling was performed in only 178 patients, of whom 80 had a defect (cohort 1) and 98 patients did not (cohort 2). However, the remaining 330 patients (cohort 3) most likely represent a mixed population, including perhaps some patients with DNA repair gene aberrations, and therefore, the results of this cohort have to be interpreted with caution. Currently, several prospective phase 2 trials (randomized and nonrandomized) evaluating carboplatin alone or in combination with docetaxel in molecularly selected patients with CRPC are recruiting.^34,35,36,37^

## Conclusions

In this multicenter international case series, platinum-based treatment was associated with promising activity in a biomarker-preselected population of patients with CRPC and DNA repair gene aberrations. These results need prospective validation, which hopefully will be provided from the currently recruiting trials in molecularly selected prostate cancer populations. Based on our analysis, platinum-containing therapy should be considered in patients with DNA repair gene aberrations, especially if access to a PARP inhibitor is not available.

## Notes

Supplement. **eTable 1. **Baseline Characteristics for All Cohorts **eTable 2. **Baseline Characteristics, by DNA Repair Defect **eFigure 1. **Median Treatment Duration, Cohort 1 vs 2 **eFigure 2. **OS by DNA Repair Alteration **eTable 3. **Multivariable Analysis OS, Cohort 1 and 2 **eTable 4. **Antitumour Activity of Platinum-Based Combination vs Monotherapy, Cohort 1 and 2 **eFigure 3. **OS of Combination vs Monotherapy, Cohort 1 vs 2 **eFigure 4. **Waterfall Plot of PSA Decline, Cohort 3 **eTable 5. **Antitumour Activity of Platinum-Based Combination vs Monotherapy, Cohort 3 **eFigure 5. **Median Treatment Duration, All Cohorts **eFigure 6. **Median OS, All Cohorts

## References

[1] Pritchard CC, Mateo J, Walsh MF, Inherited DNA-repair gene mutations in men with metastatic prostate cancer. N Engl J Med. 2016;375(5):443-453. doi:10.1056/NEJMoa1603144 27433846 PMC4986616

[2] Gillessen S, Attard G, Beer TM, Management of patients with advanced prostate cancer: the report of the Advanced Prostate Cancer Consensus Conference APCCC 2017. Eur Urol. 2018;73(2):178-211. doi:10.1016/j.eururo.2017.06.002 28655541

[3] Hager S, Ackermann CJ, Joerger M, Gillessen S, Omlin A Anti-tumour activity of platinum compounds in advanced prostate cancer—a systematic literature review. Ann Oncol. 2016;27(6):975-984. doi:10.1093/annonc/mdw156 27052650

[4] Loriot Y, Massard C, Gross-Goupil M, Combining carboplatin and etoposide in docetaxel-pretreated patients with castration-resistant prostate cancer: a prospective study evaluating also neuroendocrine features. Ann Oncol. 2009;20(4):703-708. doi:10.1093/annonc/mdn694 19179557

[5] Sternberg CN, Petrylak DP, Sartor O, Multinational, double-blind, phase III study of prednisone and either satraplatin or placebo in patients with castrate-refractory prostate cancer progressing after prior chemotherapy: the SPARC trial. J Clin Oncol. 2009;27(32):5431-5438. doi:10.1200/JCO.2008.20.1228 19805692

[6] Dasari S, Tchounwou PB Cisplatin in cancer therapy: molecular mechanisms of action. Eur J Pharmacol. 2014;740:364-378. doi:10.1016/j.ejphar.2014.07.025 25058905 PMC4146684

[7] Tutt A, Tovey H, Cheang MCU, Carboplatin in *BRCA*1/2-mutated and triple-negative breast cancer *BRCA*ness subgroups: the TNT Trial. Nat Med. 2018;24(5):628-637. doi:10.1038/s41591-018-0009-7 29713086 PMC6372067

[8] Robson M, Im SA, Senkus E, Olaparib for metastatic breast cancer in patients with a germline *BRCA* mutation. N Engl J Med. 2017;377(6):523-533. doi:10.1056/NEJMoa1706450 28578601

[9] Lin PH, Chen M, Tsai LW, Using next-generation sequencing to redefine *BRCA*ness in triple-negative breast cancer. Cancer Sci. 2020;111(4):1375-1384. doi:10.1111/cas.14313 31958182 PMC7156820

[10] Grasso CS, Wu YM, Robinson DR, The mutational landscape of lethal castration-resistant prostate cancer. Nature. 2012;487(7406):239-243. doi:10.1038/nature11125 22722839 PMC3396711

[11] Beltran H, Yelensky R, Frampton GM, Targeted next-generation sequencing of advanced prostate cancer identifies potential therapeutic targets and disease heterogeneity. Eur Urol. 2013;63(5):920-926. doi:10.1016/j.eururo.2012.08.053 22981675 PMC3615043

[12] Mateo J, Carreira S, Sandhu S, DNA-repair defects and olaparib in metastatic prostate cancer. N Engl J Med. 2015;373(18):1697-1708. doi:10.1056/NEJMoa1506859 26510020 PMC5228595

[13] Bryant HE, Schultz N, Thomas HD, Specific killing of *BRCA2*-deficient tumours with inhibitors of poly(ADP-ribose) polymerase. Nature. 2005;434(7035):913-917. doi:10.1038/nature03443 15829966

[14] Farmer H, McCabe N, Lord CJ, Targeting the DNA repair defect in *BRCA* mutant cells as a therapeutic strategy. Nature. 2005;434(7035):917-921. doi:10.1038/nature03445 15829967

[15] Fong PC, Boss DS, Yap TA, Inhibition of poly(ADP-ribose) polymerase in tumors from *BRCA* mutation carriers. N Engl J Med. 2009;361(2):123-134. doi:10.1056/NEJMoa0900212 19553641

[16] Kaufman B, Shapira-Frommer R, Schmutzler RK, Olaparib monotherapy in patients with advanced cancer and a germline *BRCA1/2* mutation. J Clin Oncol. 2015;33(3):244-250. doi:10.1200/JCO.2014.56.2728 25366685 PMC6057749

[17] Murai J, Huang SY, Das BB, Trapping of PARP1 and PARP2 by clinical PARP inhibitors. Cancer Res. 2012;72(21):5588-5599. doi:10.1158/0008-5472.CAN-12-2753 23118055 PMC3528345

[18] Cheng HH, Pritchard CC, Boyd T, Nelson PS, Montgomery B Biallelic inactivation of *BRCA2* in platinum-sensitive metastatic castration-resistant prostate cancer. Eur Urol. 2016;69(6):992-995. doi:10.1016/j.eururo.2015.11.022 26724258 PMC4909531

[19] Pomerantz MM, Spisák S, Jia L, The association between germline *BRCA2* variants and sensitivity to platinum-based chemotherapy among men with metastatic prostate cancer. Cancer. 2017;123(18):3532-3539. doi:10.1002/cncr.30808 28608931 PMC5802871

[20] Zafeiriou Z, Bianchini D, Chandler R, Genomic analysis of three metastatic prostate cancer patients with exceptional responses to carboplatin indicating different types of DNA repair deficiency. Eur Urol. 2019;75(1):184-192. doi:10.1016/j.eururo.2018.09.048 30340782 PMC6291437

[21] Mateo J, Porta N, Bianchini D, Olaparib in patients with metastatic castration-resistant prostate cancer with DNA repair gene aberrations (TOPARP-B): a multicentre, open-label, randomised, phase 2 trial. Lancet Oncol. 2020;21(1):162-174. 31806540 10.1016/S1470-2045(19)30684-9 PMC6941219

[22] Hussain M, Mateo J, Fizazi K, Saad F, Shore ND, Sandhu S, LBA12_PR: PROfound: Phase 3 study of olaparib versus enzalutamide or abiraterone for metastatic castration-resistant prostate cancer (mCRPC) with homologous recombination repair (HRR) gene alterations. Ann Oncol. 2019;30(suppl_5):v851-v934. doi:10.1093/annonc/mdz394

[23] de Bono J, Mateo J, Fizazi K, Olaparib for metastatic castration-resistant prostate cancer. N Engl J Med. 2020;382(22):2091-2102. doi:10.1056/NEJMoa1911440 32343890

[24] Editors PM; PLOS Medicine Editors Observational studies: getting clear about transparency. PLoS Med. 2014;11(8):e1001711. doi:10.1371/journal.pmed.1001711 25158064 PMC4144975

[25] Scher HI, Morris MJ, Stadler WM, ; Prostate Cancer Clinical Trials Working Group 3 Trial design and objectives for castration-resistant prostate cancer: updated recommendations from the Prostate Cancer Clinical Trials Working Group 3. J Clin Oncol. 2016;34(12):1402-1418. doi:10.1200/JCO.2015.64.2702 26903579 PMC4872347

[26] Oh WK, Tay MH, Huang J Is there a role for platinum chemotherapy in the treatment of patients with hormone-refractory prostate cancer? Cancer. 2007;109(3):477-486. doi:10.1002/cncr.22439 17186531

[27] Corn PG, Heath EI, Zurita A, Cabazitaxel plus carboplatin for the treatment of men with metastatic castration-resistant prostate cancers: a randomised, open-label, phase 1-2 trial. Lancet Oncol. 2019;20(10):1432-1443. doi:10.1016/S1470-2045(19)30408-5 31515154 PMC6858999

[28] Mateo J Biomarkers for metastatic castration-resistant prostate cancer (mCRPC): yes or no? predictive and response biomarkers towards precision medicine in mCRPC. Eur Urol Focus. 2016;2(5):465-466. doi:10.1016/j.euf.2016.06.017 28723506

[29] Abida W, Campbell D, O, Patnaik A, Sautois B, Shapiro J, 846PD Preliminary results from the TRITON2 study of rucaparib in patients (pts) with DNA damage repair (DDR)-deficient metastatic castration-resistant prostate cancer (mCRPC): updated analyses. Ann Oncol. 2019;3(suppl 5):v327-v328. doi:10.1093/annonc/mdz248.003

[30] Castro E, Romero-Laorden N, Del Pozo A, PROREPAIR-B: a prospective cohort study of the impact of germline DNA repair mutations on the outcomes of patients with metastatic castration-resistant prostate cancer. J Clin Oncol. 2019;37(6):490-503. doi:10.1200/JCO.18.00358 30625039

[31] Nombela P, Lozano R, Aytes A, Mateo J, Olmos D, Castro E *BRCA2* and other DDR genes in prostate cancer. Cancers (Basel). 2019;11(3):E352. doi:10.3390/cancers11030352 30871108 PMC6468860

[32] Abida W, Campbell D, Patnaik A, Non-*BRCA* DNA damage repair gene alterations and response to the PARP Inhibitor rucaparib in metastatic castration-resistant prostate cancer: analysis from the phase II TRITON2 Study. Clin Cancer Res. 2020;26(11):2487-2496. doi:10.1158/1078-0432.CCR-20-0394 32086346 PMC8435354

[33] Gillessen S, Attard G, Beer TM, Management of patients with advanced prostate cancer: report of the Advanced Prostate Cancer Consensus Conference 2019. Eur Urol. 2020;77(4):508-547. doi:10.1016/j.eururo.2020.01.012 32001144

[34] National Library of Medicine Docetaxel, carboplatin, and rucaparib camsylate in treating patients with metastatic castration resistant prostate cancer with homologous recombination DNA repair deficiency. Updated September 9, 2020. Accessed September 29, 2020. https://clinicaltrials.gov/ct2/show/NCT03442556?term=NCT03442556&draw=2&rank=1

[35] National Library of Medicine BRcA deficient prostate cancer treated with carboplatin or docetaxel (BRACeD). Updated August 7, 2020. Accessed September 29, 2020. https://clinicaltrials.gov/ct2/show/NCT04038502?term=NCT04038502&draw=2&rank=1

[36] National Library of Medicine Docetaxel and carboplatin in treating patients with metastatic, castration resistant prostate cancer containing inactivated genes in the *BRCA 1/2* pathway. Updated June 16, 2020. Accessed September 29, 2020. https://clinicaltrials.gov/ct2/show/NCT02598895?term=NCT02598895&draw=2&rank=1

[37] National Library of Medicine Trial evaluating the efficacy of carboplatin in metastatic prostate cancer with gene alterations in the homologous recombination pathway (PRO-CARBO). Updated February 17, 2020. Accessed September 29, 2020. https://clinicaltrials.gov/ct2/show/NCT03652493?term=NCT03652493&draw=2&rank=1

