# Errors in Byline, Affiliations, and Conflict of Interest Disclosures

**DOI:** 10.1001/jamanetworkopen.2020.29176

**Published:** 2020-11-18

In the Original Investigation titled “Activity of Platinum-Based Chemotherapy in Patients With Advanced Prostate Cancer With and Without DNA Repair Gene Aberrations,”^1^ published on October 28, 2020, there were errors in the byline, affiliations, and Conflict of Interest Disclosures. Dr Conteduca’s and Dr Lavaud’s names appeared incorrectly in the byline. Dr Conteduca is not affiliated with the Department of Medical Oncology, Weill Cornell Medicine. Drs Mehra and Kuppen’s affiliation should have read Department of Medical Oncology, Radboud University Medical Center, Nijmegen, the Netherlands. Dr Conteduca’s conflict of interest disclosure was omitted. This article has been corrected.^1^
