# Supplementary material for: Activity of Platinum-Based Chemotherapy in Patients With Advanced Prostate Cancer With and Without DNA Repair Gene Aberrations
Source: JAMA Netw Open. 2020 Oct 28;3(10):e2021692. doi: 10.1001/jamanetworkopen.2020.21692 (PMC7593810; doi:10.1001/jamanetworkopen.2020.21692)
Supplement: Supplement. — eTable 1. Baseline Characteristics for All Cohorts eTable 2. Baseline Characteristics, by DNA Repair Defect eFigure 1. Median Treatment Duration, Cohort 1 vs 2 eFigure 2. OS by DNA Repair Alteration eTable 3. Multivariable Analysis OS, Cohort 1 and 2 eTable 4. Antitumour Activity of Platinum-Based Combination vs Monotherapy, Cohort 1 and 2 eFigure 3. OS of Combination vs Monotherapy, Cohort 1 vs 2 eFigure 4. Waterfall Plot of PSA Decline, Cohort 3 eTable 5. Antitumour Activity of Platinum-Based Combination vs Monotherapy, Cohort 3 eFigure 5. Median Treatment Duration, All Cohorts eFigure 6. Median OS, All Cohorts [file jamanetwopen-e2021692-s001.pdf]

Supplemental Online Content

Schmid S, Omlin A, Higano C, et al. Activity of platinum-based chemotherapy in patients with advanced prostate cancer with and without DNA repair gene aberrations. *JAMA Netw Open*. 2020;3(10):e2021692. doi:10.1001/jamanetworkopen.2020.21692

eTable 1. Baseline Characteristics for All Cohorts

eTable 2. Baseline Characteristics, by DNA Repair Defect

eFigure 1. Median Treatment Duration, Cohort 1 vs 2

eFigure 2. OS by DNA Repair Alteration

eTable 3. Multivariable Analysis OS, Cohort 1 and 2

eTable 4. Antitumour Activity of Platinum-Based Combination vs Monotherapy, Cohort 1 and 2

eFigure 3. OS of Combination vs Monotherapy, Cohort 1 vs 2

eFigure 4. Waterfall Plot of PSA Decline, Cohort 3

eTable 5. Antitumour Activity of Platinum-Based Combination vs Monotherapy, Cohort 3

eFigure 5. Median Treatment Duration, All Cohorts

eFigure 6. Median OS, All Cohorts

This supplemental material has been provided by the authors to give readers additional information about their work.

© 2020 Schmid S et al. *JAMA Network Open.*

eTable 1. Baseline Characteristics for All Cohorts

	Cohort 1: DNA repair defects assessed, present (N = 80)	Cohort 2: DNA repair defects assessed, not present (N = 98)	Cohort 3: DNA repair defects not assessed (N = 330)	P (all 3 cohorts)	P (cohort 1 vs. 2)
	**N (%)**	**N (%)**	**N (%)**		
**At diagnosis**					
Gleason Score (median, range)	8 (6-10), 8NA	8 (5-10), 10NA	8 (5-10), 22NA	0.7	0.6
PSA (median, range)	33 (2.1-1759), 5NA	17.5 (1.1-1530), 15NA	18.4 (0.7-7577), 57NA	0.3	0.2
Age at diagnosis (median, range)	59 (37-78), 0NA	60 (46-80), 1NA	61 (40-88), 2NA	0.1	0.2
**Metastatic situation (de-novo versus after local treatment)**					
• After primary local therapy	38 (47.5%)	58 (59.2%)	196 (59.4%)	0.2	0.1
• At diagnosis	42 (52.5%)	40 (40.8%)	134 (40.6%)		
**mCRPC treatment before platinum-based chemotherapy**					
Abiraterone	33 (23.6%)	47 (27.3%)	97 (19.1%)	0.001	0.5
Cabazitaxel	22 (15.7%)	30 (17.4%)	79 (15.6%)	0.4	0.8
Docetaxel	40 (28.6%)	39 (22.7%)	205 (40.4%)	<.0001	0.2
Enzalutamide	29 (20.7%)	39 (22.7%)	88 (17.3%)	0.02	0.7
PARP inhibitor	7 (5%)	1 (0.6%)	0	<.0001	0.04
Radium-223	9 (6.4%)	16 (9.3%)	38 (7.5%)	0.4	0.5

© 2020 Schmid S et al. *JAMA Network Open.*

eTable 2. Baseline Characteristics, by DNA Repair Alteration

	BRCA 1 (n=3)	BRCA 2 (N = 44)	ATM (N=12)	Other (n=21)	P
**At diagnosis**					
Gleason Score (median, range)	9 (8-10), 0NA	9 (6-10), 3NA	7 (6-9), 3NA	8 (6-10), 2NA	0.1
PSA (median, range)	148 (6.5-199), 0NA	17.1 (2.1-1440), 4NA	12.8 (4-57.42), 1NA	110 (6.9-1759), 0NA	**0.01**
Age at diagnosis (median, range)	65 (64-67), 0NA	60.5 (37-78), 0NA	55 (46-69), 0NA	57 (44-67), 0NA	0.09
**Metastatic situation (de-novo versus after local treatment)**					
• After primary local therapy	1 (33.3%)	23 (52.3%)	5 (41.7%)	9 (42.9%)	0.8
• At diagnosis	2 (66.7%)	21 (47.7%)	7 (58.3%)	12 (57.1%)	
**mCRPC treatment before platinum-based chemotherapy**					
Abiraterone	1 (33.3%)	19 (43.2%)	5 (41.7%)	8 (38.1%)	>0.99
Cabazitaxel	0 (0%)	8 (18.2%)	4 (33.3%)	10 (47.6%)	0.07
Docetaxel	2 (66.7%)	17 (38.6%)	6 (50%)	15 (71.4%)	0.08
Enzalutamide	1 (33.3%)	17 (38.6%)	7 (58.3%)	4 (19%)	0.1
PARP inhibitor	0 (0%)	5 (11.4%)	2 (16.7%)	0 (0%)	0.3
Radium-223	1 (33.3%)	6 (13.6%)	2 (16.7%)	0 (0%)	0.1

© 2020 Schmid S et al. *JAMA Network Open.*

eFigure 1. Median Treatment Duration, Cohort 1 vs 2

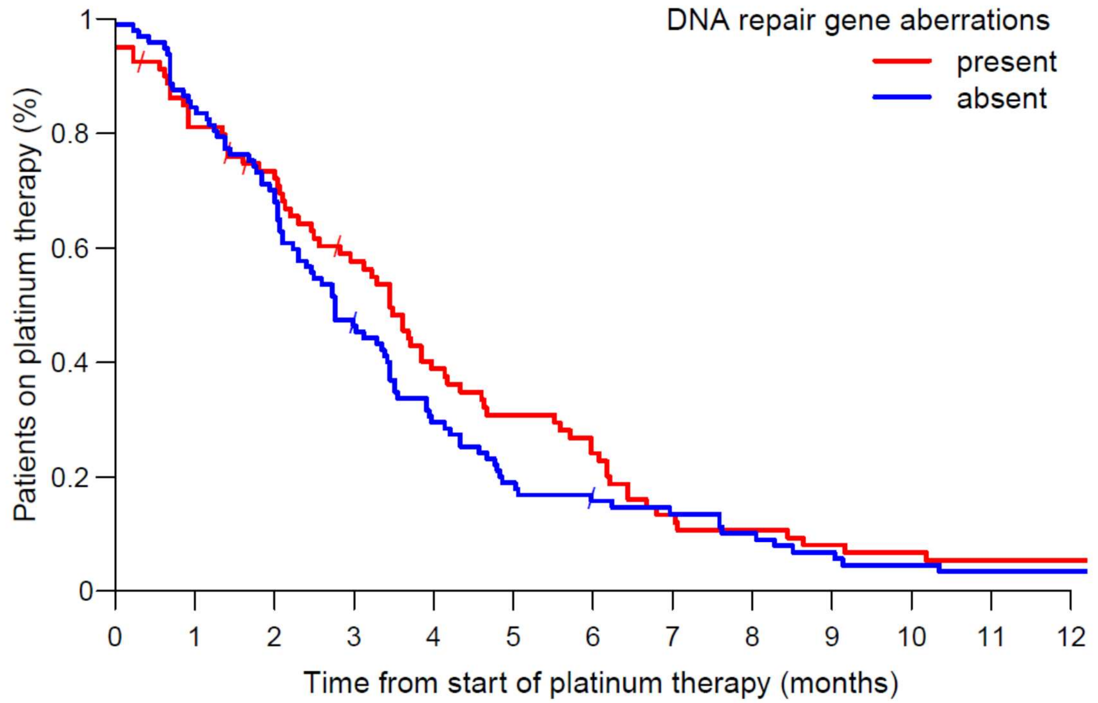

No. at risk													
present	76	64	56	43	29	23	18	10	8	6	5	4	4
absent	96	82	68	44	28	18	14	12	9	6	4	3	3

© 2020 Schmid S et al. *JAMA Network Open.*

eFigure 2. OS by DNA Repair Alteration

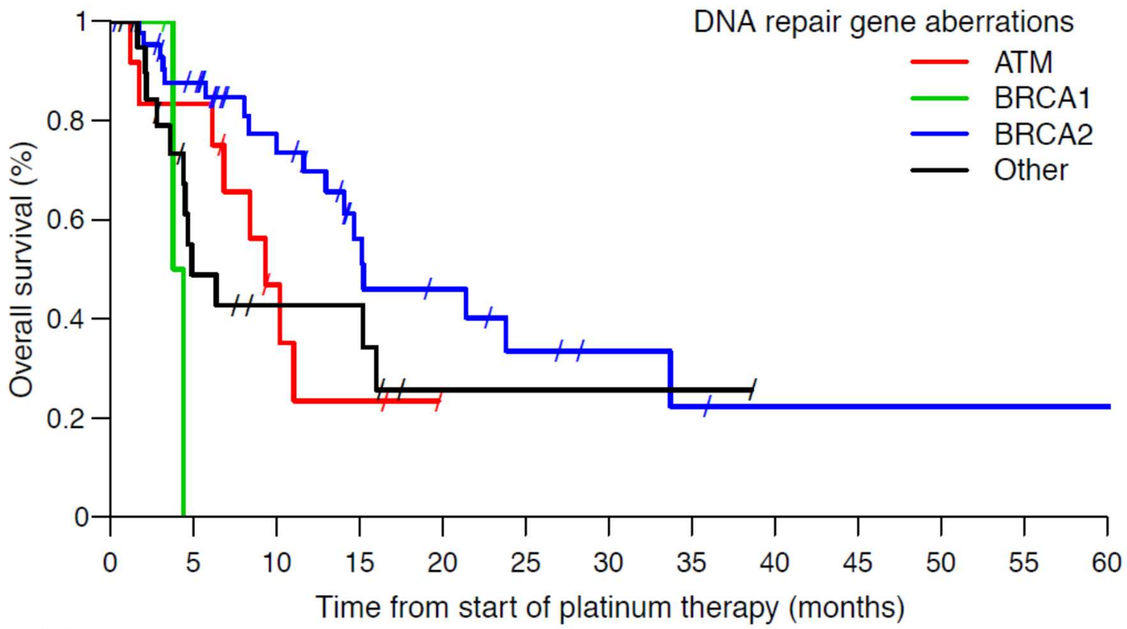

No. at risk

ATM	12	10	4	2	0	0	0	0	0	0	0	0	0
BRCA1	3	0	0	0	0	0	0	0	0	0	0	0	0
BRCA2	44	32	20	11	8	5	3	2	1	1	1	1	1
Other	21	8	5	5	1	1	1	1	0	0	0	0	0

© 2020 Schmid S et al. *JAMA Network Open.*

eTable 3. Multivariable Analysis OS, Cohort 1 and 2
N (used) = 121 (only patients with all available data included)
OS (overall survival), vs. (versus), DNA (deoxyribonucleic acid)

Variable	HR	95% CI	p-value
Age at diagnosis (years)	0.984	0.950 - 1.018	0.4
Visceral metastases Yes vs. No	0.834	0.298 - 2.338	0.7
DNA repair gene aberrations: Yes vs. No	0.753	0.466 - 1.217	0.2
Type of platinum therapy combination vs. monotherapy	0.516	0.310 - 0.859	**0.01**
Treatment line (>1 vs. 1)	0.800	0.499 - 1.280	0.4

© 2020 Schmid S et al. *JAMA Network Open.*

eTable 4. Antitumour Activity of Platinum-Based Combination vs Monotherapy, Cohort 1 and 2

	Platinum monotherapy		p-value	Platinum combination therapy		p-value
	Cohort 1 (n= 31)	Cohort 2 (n= 11)		Cohort 1 (n= 49)	Cohort 2 (n= 87)	
**PSA decline ≥50% from baseline**	10 (32.3%)	3 (27.3%)	>0.99	23 (46.9%)	23 (26.4%)	0.02
**Soft tissue response**	14 (45.2%)	3 (27.3%)	0.5	14 (28.6%)	18 (20.7%)	0.3
**Median OS from start of platinum therapy (months)**	6.4 (IQR 3.6-15)	6.7 (IQR 3.5-15)	0.8	15 (IQR 10-34)	9.8 (IQR 6.1-19)	0.02

© 2020 Schmid S et al. *JAMA Network Open.*

eFigure 3. OS of Combination vs Monotherapy, Cohort 1 vs 2

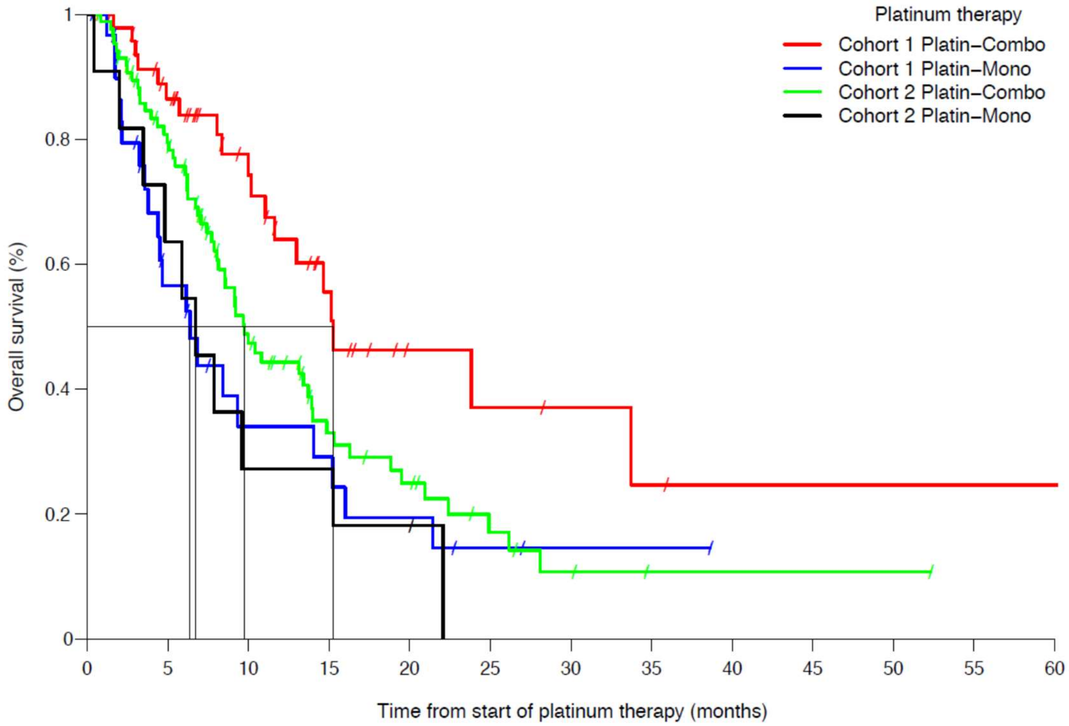

No. at risk													
Cohort 1 Platin–Combo	49	36	22	12	5	4	3	2	1	1	1	1	1
Cohort 1 Platin–Mono	31	14	7	6	4	2	1	1	0	0	0	0	0
Cohort 2 Platin–Combo	87	63	32	17	12	6	3	1	1	1	1	0	0
Cohort 2 Platin–Mono	11	7	3	3	2	0	0	0	0	0	0	0	0

© 2020 Schmid S et al. *JAMA Network Open.*

eFigure 4. Waterfall Plot of PSA Decline, Cohort 3

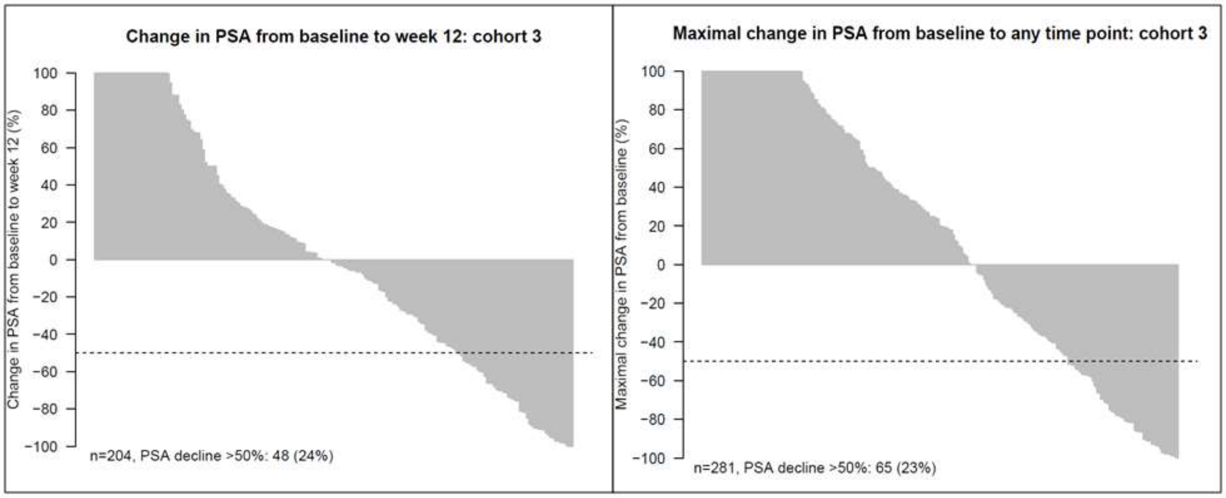

© 2020 Schmid S et al. *JAMA Network Open.*

eTable 5. Antitumour Activity of Platinum-Based Combination vs Monotherapy, Cohort 3

DNA (deoxyribonucleic acid), PSA (prostatic specific antigen), IQR (interquartile range), NA (not available), m (months)

	**Overall Cohort 3 (N = 330)**	**Platinum Monotherapy (N=58)**	**Platinum Combination therapy (N=272)**
	**N (%)**		
**PSA decline on platinum therapy**			
PSA decline ≥ 50% from baseline	81 (28.5%)	12 (25.0%)	69 (29.2%)
NA	46	10	36
**Soft tissue response on platinum therapy**			
No	147 (79.5%)	36 (87.8%)	111 (77.1%)
Yes (investigator assessed)	38 (20.5%)	5 (12.2%)	33 (22.9%)
NA	145	17	128
**Time on treatment and survival**			
Median time on platinum therapy (m, IQR)	3 (IQR 1.7 to 4.6), 13NA	2.4 (IQR 0.85-4)	3 (IQR 1.9-4.6)
Median OS from start of platinum therapy (m, IQR)	10 (IQR 5.7 to 18), 4NA	8.1 (IQR 4.7-13)	10 (IQR 6.1-18)

© 2020 Schmid S et al. *JAMA Network Open.*

eFigure 5. Median Treatment Duration, All Cohorts

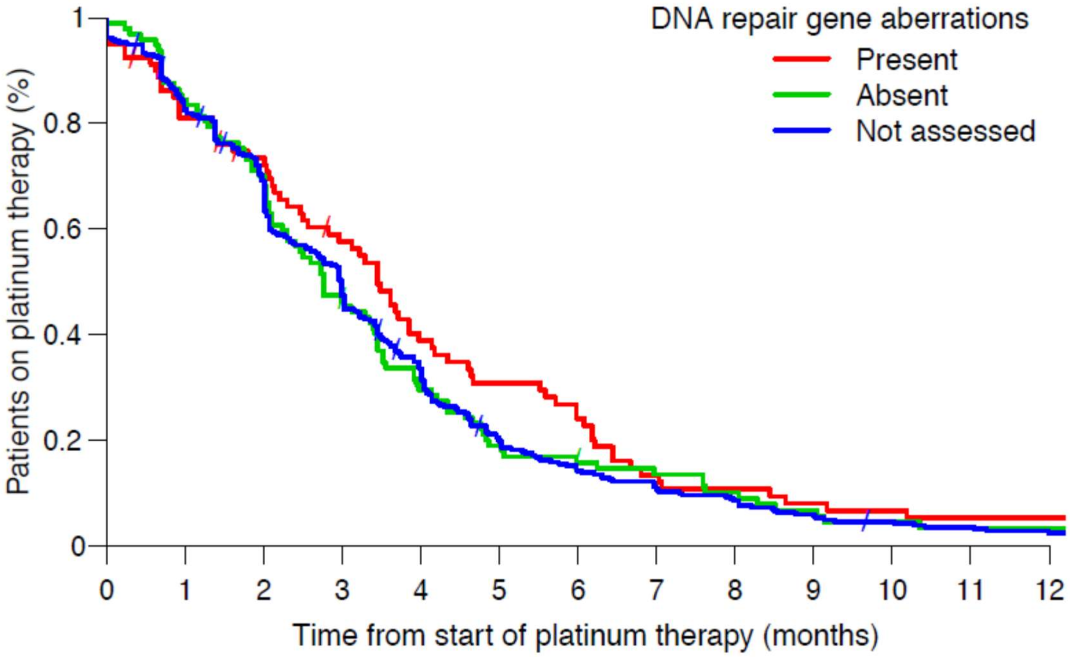

No. at risk													
Present	76	64	56	43	29	23	18	10	8	6	5	4	4
Absent	96	82	68	44	28	18	14	12	9	6	4	3	3
Not assessed	305	260	216	148	103	60	43	32	26	18	13	10	7

© 2020 Schmid S et al. *JAMA Network Open.*

eFigure 6. Median OS, All Cohorts

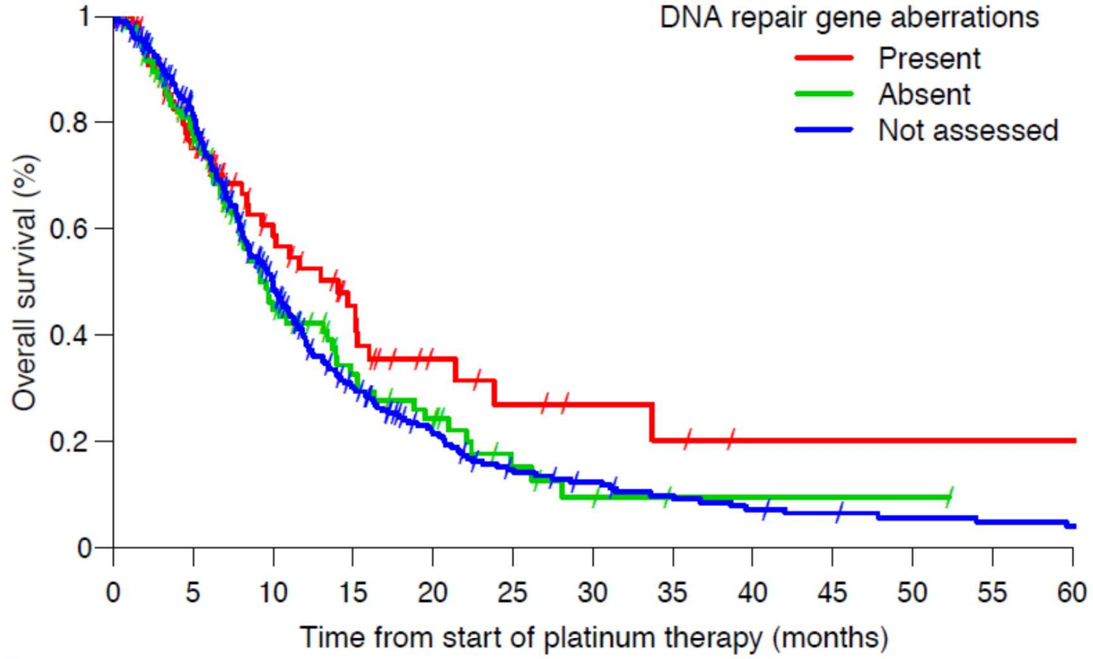

No. at risk													
Present	80	50	29	18	9	6	4	3	1	1	1	1	1
Absent	98	70	35	20	14	6	3	1	1	1	1	0	0
Not assessed	325	242	133	72	42	26	20	15	11	9	7	6	5

© 2020 Schmid S et al. *JAMA Network Open.*
